# In-depth immune cellular profiling reveals sex-specific associations with frailty

**DOI:** 10.1186/s12979-020-00191-z

**Published:** 2020-06-23

**Authors:** Leonard Daniël Samson, A. Mieke H. Boots, José A. Ferreira, H. Susan J. Picavet, Lia G. H. de Rond, Mary-lène de Zeeuw-Brouwer, W. M. Monique Verschuren, Anne-Marie Buisman, Peter Engelfriet

**Affiliations:** 1 National Institute of Public Health and the Environment, Bilthoven, 3722 BA Netherlands; 2 Department of Rheumatology and Clinical Immunology, University Medical Center Groningen, University of Groningen, Groningen, 9727 Netherlands; 3 Julius Center for Health Sciences and Primary Care, University Medical Center Utrecht, Utrecht University, Utrecht, 3553 Netherlands

**Keywords:** Frailty, Immune cellular profiling, Immunosenescence, Sex-specific immune profile, Immune homeostasis, Healthy aging

## Abstract

**Background:**

With advancing age, the composition of leukocyte subpopulations in peripheral blood is known to change, but how this change differs between men and women and how it relates to frailty is poorly understood. Our aim in this exploratory study was to investigate whether frailty is associated with changes in immune cell subpopulations and whether this differs between men and women. Therefore, we performed in-depth immune cellular profiling by enumerating a total of 37 subpopulations of T cells, B cells, NK cells, monocytes, and neutrophils in peripheral blood of 289 elderly people between 60-87 years of age. Associations between frailty and each immune cell subpopulation were tested separately in men and women and were adjusted for age and CMV serostatus. In addition, a random forest algorithm was used to predict a participant’s frailty score based on enumeration of immune cell subpopulations.

**Results:**

In the association study, frailty was found to be associated with increased numbers of neutrophils in both men and in women. Frailer women, but not men, showed higher numbers of total and CD16^-^ monocytes, and lower numbers of both CD56^+^ T cells and late differentiated CD4^+^ TemRA cells. The random forest algorithm confirmed all the findings of the association studies in men and women. In men, the predictive accuracy of the algorithm was too low (5.5%) to warrant additional conclusions on top of the ones derived from the association study. In women however, the predictive accuracy was higher (23.1%), additionally revealing that total T cell numbers and total lymphocyte numbers also contribute in predicting frailty.

**Conclusions:**

In-depth immune cellular profiling revealed consistent associations of frailty with elevated numbers of myeloid cell subpopulations in both men and women. Furthermore, additional associations were found between frailty and lower numbers of some T cell subpopulations, in women only. Thus, our study indicates sex-specific associations of immune subpopulations with frailty. We hope that our study will prompt further investigation into the sex-specific immune mechanisms associated with the development of frailty.

## Background

While the biological process of aging is inevitable, some people remain healthy until an advanced age while others suffer from age-related diseases early in life. The reasons for these vastly different aging patterns are still poorly understood. To protect the body from damage normally associated with aging, a balanced immune system is needed [1], with different subpopulations of immune cells working in close harmony. One telling sign of a disturbed immune balance is a state of chronic low-grade inflammation, which may be revealed by measuring biomarkers such as C-reactive protein [1–3]. It remains a challenge, however, to identify other biomarkers of the immune system that signal or explain differences in aging patterns.

Both innate and adaptive immune cell lineages are essential for a proper functioning of the immune system. It is well-established that the composition of the immune cell repertoire—that is the relative and absolute abundances of the various subpopulations of immune cells—changes with age [4]. However, it is largely unknown how changes in the immune cellular composition differ between individuals who age in good health from those who become frail at a relatively early age. Several studies have been performed on this subject, but some of these were done in the extreme elderly of 85 years old or above [5], while others focused on a limited set of lymphocyte phenotypes [6–8]. Importantly, few studies have been performed enumerating a comprehensive set of immune phenotypes in freshly drawn whole blood samples.

Recently, we investigated associations between frailty and absolute numbers of the major immune cell populations in fresh whole blood. In that study, we detected associations between frailty and numbers of granulocytes and monocytes [3]. However, the complex functioning of the immune system involves a range of specialized functions that are mediated by various myeloid cells and lymphocytes. It is therefore to be expected that shifts in the major immune cell populations are accompanied by less visible, yet potentially important, changes in their subpopulations that are functionally distinct. This hypothesis prompted us to extend our previous study with an in-depth analysis of various immune cell subpopulations in relation to frailty.

Since men and women tend to age differently [9], we deemed it important to consider differences between the sexes. Furthermore, cytomegalovirus (CMV) serostatus should be taken into account, because chronic CMV infection is well known to impact numbers of immune cell subpopulations [10, 11].

Thus, our main goal in this exploratory study was to investigate how general health, expressed in terms of a frailty index, is associated with various immune cell subpopulations in peripheral blood of older adults. Our secondary goal was to explore how these cell populations differ between the sexes or according to CMV serostatus. In order to do so, we employed extensive immune profiling, enumerating 37 immune cell subpopulations and quantifying the expression of several surface markers on immune cells in fresh whole blood samples from 289 older people aged between 60-87 years that were selected from among the participants in an ongoing cohort study in the Netherlands [12, 13].

## Methods

### DCS subcohort selection

A subcohort was selected from the Doetinchem cohort study (DCS) [12, 13]. Details on this subcohort and its selection have been described previously [3]. Briefly, the study involved 289 active DCS participants, aged 60-87 years, who were selected as an age- and sex- stratified sample, with selection of equal numbers of the healthiest, intermediate, and frailest participants. By healthiest/frailest we mean those belonging to the 15% individuals with the lowest/highest frailty index score (see below for the definition of frailty index) compared to their age- and sex-matched peers. By intermediate we mean the remaining 70% of the DCS participants.

### Frailty index

A frailty index was constructed based on previous studies [5, 14–16] and was adapted to the data available in the DCS; it was recently validated within the DCS by [3]. This frailty index incorporates 36 possible “health deficits” such as presence of (a particular) chronic disease or reduced physical functioning. The values of the frailty index are restricted to lie between zero and one, zero representing the ‘best’ (0 out of 36 deficits present) and one representing the ‘worst’ (all 36 deficits present) health status. Since it has 36 categories, this frailty index is by approximation a continuous variable. Using the index, a frailty ‘score’ was calculated for each individual, based on data collected during the DCS assessment round 6 (2012-2017). Twelve out of 289 individuals who participated in our DCS subcohort, had not participated in DCS assessment round 6 and thus, their frailty index scores were missing.

### Whole blood lymphocyte phenotyping

Fresh whole blood samples from the DCS subcohort participants were collected between August 2016 and March 2017, and were processed and analyzed within 6 hours on a 4-laser LSRII Fortessa X20 flow cytometer (BD Biosciences) for absolute numbers of leukocyte subpopulations (cell counts *μ*L^-1^). Two labeled antibody panels per participant were used with a lyse-no-wash protocol, one in a TruCOUNT$^{\circledR }$ tube (BD Biosciences) and one in a common (Falcon) tube. In both panels we used the fluorochrome-conjugated antibodies CD3(UCHT1)-BV711 (BD) and CD27(M-T271)-BV421 (Biolegend). In the TruCOUNT$^{\circledR }$ tube, we additionally used CD56(B159)-APC, CD8(SK1)-FITC, CD16(B73.1)-PE, CD4(SK3)-PerCPCy5.5, IgD(ia6-2)-BB515, CD38(HB7)-APC-H7, HLA-DR(G46.6)-PECF594 (all BD Biosciences), CD19(J3-119)-PECy7 (Beckman Coulter), and CD45(GA90)-OC515 (Cytognos). In the second tube we additionally used the following fluorochrome-conjugated antibodies: CD127(hIL-7R-M21)-PE, CD25(2A3)-BB515, CCR7(150503)-PECF594, CD28(CD28.2)-PerCPCy5.5, CD8(SK1)-APC-H7 (all BD Biosciences), CD4(RPA-T4)-BV510, CD45RA(HI100)-BV650 (all Biolegend), and CXCR5(51505)-APC (R&D Systems). Absolute cell numbers in the Falcon tubes were calculated by using the CD3 T cell ratio between both tubes and the bead count in the TruCOUNT$^{\circledR }$ tube.

For phenotype definitions and gating strategies, see Table S1 and Figures S1–S3. Neutrophils were gated as CD45 and SSC^BRIGHT^ and CD45^DIM^ and were additionally analyzed not only regarding cell numbers but also with respect to CD16 expression. CD16 is usually expressed on the surface of neutrophils [17] and is seen as a neutrophil maturation marker [18]. Lower expression of CD16 by neutrophils was seen in several diseases and in states of neutropenia [19]. Monocytes were gated as SSC^DIM^CD45^DIM^ and, to ensure that B cells or T cells did not contaminate the gate, as CD3^-^CD19^-^. Monocytes were further sub-classified into CD16^-^ and CD16^+^ monocytes and were additionally analyzed based on the expression of HLA-DR and CD38, since HLA-DR expression on monocytes is thought to be lower [20] and CD38 expression higher [21] in inflammatory conditions. NK cells were gated and subdivided based on their CD16 and CD56 expression [22]. For memory T cells and regulatory T cells, gating was done as described previously [23–25] and was performed similarly in both CD4^+^ and CD8^+^ T cells. In short, CCR7^+^ CD4^+^/CD8^+^ T cells were classified as either naïve (CD45RA^+^CCR7^+^) or central memory (CCR7^+^CD45RA^-^) T cells. CCR7^-^ CD4^+^/CD8^+^ T cells were divided into effector memory T cells (Tem, CCR7^-^CD45RA^-^) and effector memory T cells re-expressing CD45RA (TemRA, CCR7^-^CD45RA^+^) T cells. Finally, these T cells were further subclassified into early (CD27^+^CD28^+^) and late stage (CD27^-^CD28^-^) Tem or TemRA cells. The B cell subpopulations were defined by means of CD19, CD27, and CD38 expression [24], with naïve B cells defined as CD19^+^CD38^DIM^CD27^-^ and memory B cells as CD19^+^CD38^DIM^CD27^+^ (Figure S3 and Table S1). As an additional analysis, we also calculated proportions of the immune cell subpopulations. Proportions were expressed as the percentage of their major cell lineage (T cells, B cells, NK cells, or monocytes) or, for all CD4^+^ and CD8^+^ subpopulations, as the percentage of CD4^+^ and CD8^+^ T cells, respectively.

Some subpopulations were not clearly distinguishable from debris and the corresponding data were excluded from analysis. This occurred for the general lymphocyte subpopulation in four participants, for the monocyte subpopulations in three participants, and for both the lymphocyte and monocyte subpopulations in three participants. Unwanted fluorochrome excitation spillover was automatically corrected for by using BDComp beads (BD Biosciences). Gating of cellular subpopulations was performed in FlowJo V10 (FlowJo company).

### Anti-cytomegalovirus antibodies

IgG antibodies against CMV were quantified using a multiplex immunoassay (MIA) that was developed in-house and was based on a commercially available Cytomegalovirus IgG ELISA kit (EUROIMMUN, Germany) [26]. A cutoff for CMV-seropositivity was calculated by first pooling IgG CMV antibody concentrations of people from multiple cohorts [3, 27–29], with all concentrations measured using the same in-house developed assay (n=1415, age range: 4-89 years). With these pooled concentrations, we used mixture modeling [30] to define two distributions, representing respectively CMV-seronegative and CMV-seropositive individuals. The CMV-seropositivity cutoff was defined as the intersection between the two distributions, as described previously for varicella zoster virus [31].

### Statistical methods

#### Overall plan of analysis

The aim of the statistical analyses was to investigate the relation between the various leukocyte subpopulations and the characteristics of interest, namely sex, CMV status and frailty, the latter being our main focus. In order to analyze these relationships, we used bivariate analyses in association studies, which are described in detail below. We also performed multivariate analyses to study the collective effect of the leukocyte subpopulations on frailty. Both in the bivariate and in the multivariate analysis, we used non-parametric methods because these depend on fewer assumptions regarding the underlying data such as normality of distribution, and are thus less prone to lead to invalid conclusions. For the multivariate analysis, with frailty as dependent variable, we used the random forest algorithm. All analyses were performed using R version 3.6.0 [32] and several tidyverse packages [33]. Figures were generated with the R packages ggplot2 (part of tidyverse) and cowplot [34].

#### Association studies

In the bivariate association studies, we used the permutation version of the Wilcoxon-Mann-Whitney test to analyze the associations between each leukocyte subpopulation and sex [35]. We used the same test to analyze the associations between each leukocyte subpopulation and CMV serostatus, separately for men and women. The permutation version of the Spearman test was used to analyze the associations between each of the leukocyte subpopulations with frailty, also separately for men and women [35]. These tests (both implemented in the coin package of R) are based on ranking the variables in order of magnitude. *P*-values were calculated using simulations, for which we set the number of simulations at 10^8^. In order to take into account potential confounding due to age or CMV status, we stratified the analyses using a block design. The “blocks” were defined based on a division according to age into four age groups for all analyses, and also according to CMV status into positive and negative status for the associations between the sexes and for the ones with frailty. To quantify the strength of the correlations with frailty, we calculated the weighted average of Spearman’s rho per block. In order to account for multiple testing, we implemented the Benjamini Hochberg method [36] separately for each association study, applying a False Discovery Rate (FDR) of 15%.

#### Random forest analysis

We complemented the association study regarding frailty with a multivariate analysis. The cell subpopulations were entered into a prediction study using the random forest algorithm [37]. That is, a participant’s frailty index was predicted with these variables together with age, CMV serostatus and date of flow cytometry measurement. The random forest algorithm ranks the independent variables according to their ‘importance’, or influence, in predicting the dependent variable (frailty). The importance of a variable is expressed in terms of the percentage increase in mean-square error (MSE) when the effect of that variable is removed. The overall performance of the prediction analysis was assessed in terms of the percentage explained variance. The frailty index score was transformed by taking the square root, in order to obtain a more symmetric distribution. In order to obtain a qualitative idea of the relationships between the most important predictor variables and the frailty index, partial dependence plots were created with the pdp R package [38], showing the magnitude and direction of the effect of the respective variables on frailty.

## Results

### Study population

The study population consisted of 145 men and 144 women. Average time between blood sample collection and frailty index measurement was 1.6 years (95%CI: 1.4-1.8 years, min: 2 days, max: 4.25 years). Since five men and seven women did not participate in the latest DCS assessment round and therefore had a missing frailty index score for that round, these participants were removed from the analyses with frailty.

### Associations between leukocyte subpopulations and sex

**Fig. 1:**
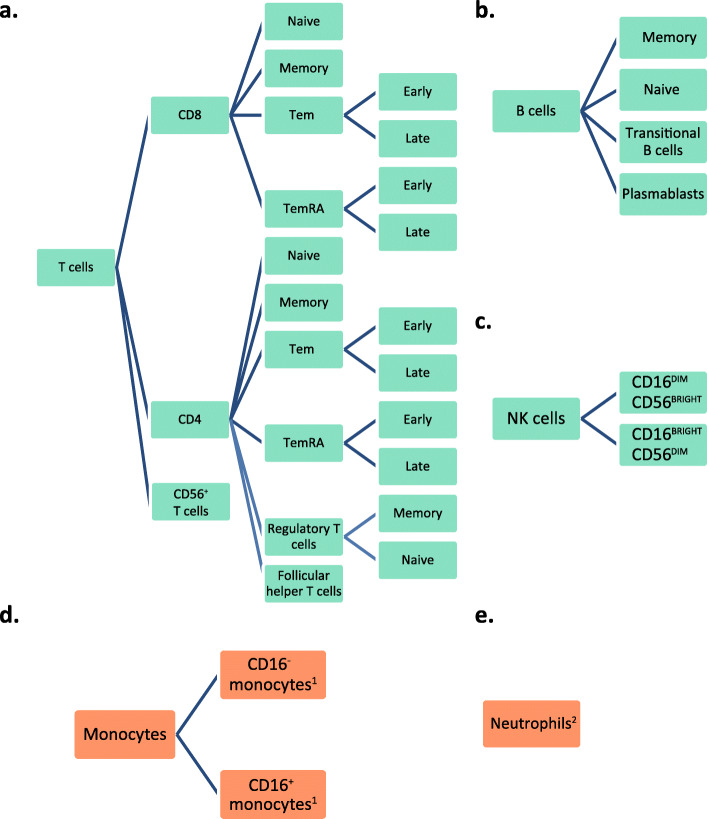
Subpopulations of **a** T cells, **b** B cells, **c** NK cells, **d** Monocytes, and **e** Neutrophils, of which absolute cell numbers were determined. CM: central memory (CD4 or CD8 positive) T cells. Tem: effector memory (CD4 or CD8 positive) T cells. TemRA: effector memory T cells re-expressing CD45RA. See Table S1 for definition of all phenotypes. ^1^Additionally analyzed on expression of CD38 and HLA-DR. ^2^Additionally analyzed on expression of CD16

**Fig. 2:**
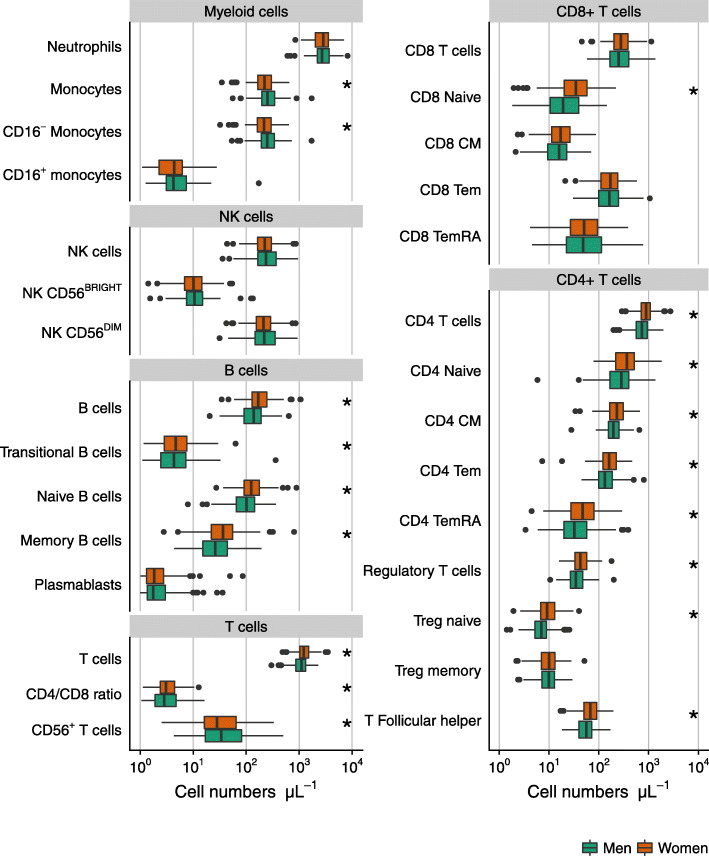
The numbers of cells per leukocyte subpopulations in men (n=145) and women (n=144) above 60 years of age. The boxplots show median values with interquartile range. CM: central memory (CD4 or CD8 positive) T cells. Tem: effector memory (CD4 or CD8 positive) T cells. TemRA: effector memory T cells re-expressing CD45RA. *: Outcomes of tests of the association between leukocyte numbers and sex (adjusted for age and CMV serostatus) which passed the Benjamini-Hochberg method at a false discovery rate of 15%

**Fig. 3:**
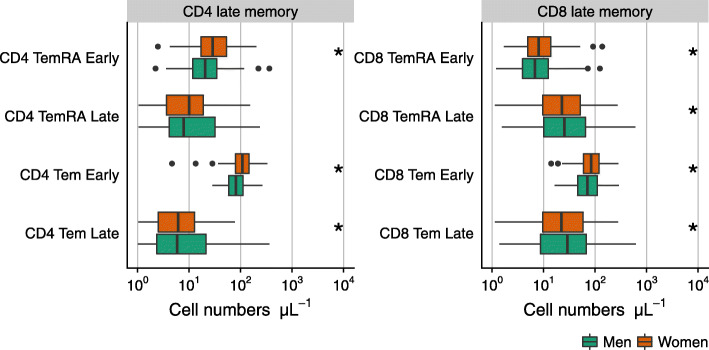
The numbers of CD4 and CD8 Tem and TemRA cells in men (n=145) and women (n=144) above 60 years of age. The boxplots show median values with interquartile range. CM: central memory (CD4 or CD8 positive) T cells. Tem: effector memory (CD4 or CD8 positive) T cells. TemRA: effector memory T cells re-expressing CD45RA. *: Outcomes of tests of the association between leukocyte numbers and sex (adjusted for age and CMV serostatus) which passed the Benjamini-Hochberg method at a false discovery rate of 15%

An overview of immune cell phenotypes considered in this study is given in Fig. 1, all of them measured in peripheral blood. Most of the analyzed subpopulations belong to the T cell lineage, as in the literature these have been reported to be affected by aging and CMV [10, 11, 39]. Neutrophils accounted for the highest median numbers in peripheral blood of both men and women, followed by T cells (Fig. 2). Women had lower numbers of CD16^-^ (classical) monocytes but higher numbers of B cells and T cells than men. Cell numbers were higher in women for almost all measured B cell subpopulations, namely for transitional B cells, naïve B cells, and memory B cells. The higher numbers of T cells in women were mainly due to higher CD4^+^ T cell numbers. Further subsetting revealed higher concentrations of almost all CD4 T cell subpopulations (naïve CD4 T cells, central memory CD4 T cells, CD4 Tem and TemRA cells, (naïve) regulatory T cells, and Follicular helper T cells) in peripheral blood of women. This pattern was also seen in early differentiated CD4 subpopulations but, in contrast, not in late differentiated CD4 Tem cells which showed lower numbers in women (Fig. 3). The association of lower late differentiated CD4 Tem numbers in women would have been missed without stratification for CMV serostatus since the association was mainly seen in CMV seropositive women (data not shown). Furthermore, we also observed lower numbers of CD56^+^ T cells in women (Fig. 2). While total numbers of CD8^+^ T cells were not found to be different between the sexes, CD8 naïve T cell numbers were observed to be higher in women (Fig. 2). More detailed phenotyping of CD8 T cell subpopulations revealed higher numbers of early differentiated CD8 Tem and TemRA cells in women than in men, but lower numbers of late differentiated CD8 Tem and TemRA cells (Fig. 3).

### Associations between leukocyte subpopulations and CMV serostatus

CMV seropositivity was associated with higher numbers of T cells and CD56^+^ T cells in men and in women (Table S2). Numbers of CD8^+^ T cells were observed to be higher in CMV seropositive (CMV^+^) men and CMV^+^ women, which was mainly due to higher numbers of late stage CD8^+^ Tem and TemRA cells. Numbers of CD4^+^ T cells were observed to be higher in CMV^+^ men but not in CMV^+^ women. However, late stage CD4^+^ Tem and TemRA cells were higher in CMV^+^ men and CMV^+^ women. Also, the CD4/CD8 ratio was lower in CMV seropositive individuals of both sexes, indicating that the higher T cell numbers in CMV^+^ participants was due to the higher numbers of CD8^+^ T cells rather than to the CD4^+^ T cells. In men, CMV seropositivity was also associated with higher numbers of follicular helper T cells and with lower numbers of monocytes due to lower numbers of CD16^-^ (classical) monocytes. These classical monocytes also showed higher CD38 expression.

### Relationship of frailty with leukocyte subpopulations

In the association study, an association between frailty and higher neutrophil numbers was found in both men and women (tables S3 and S4; *ρ*=0.25 and *ρ*=0.4, respectively), while other associations were observed only in women. Frailer women, but not men, showed higher monocyte numbers (*ρ*=0.23), in particular CD16^-^ classical monocyte numbers (*ρ*=0.24). In addition, frailer women also showed lower CD56^+^ T cell numbers (*ρ*=-0.20) and lower CD4^+^ TemRA cell numbers (*ρ*=-0.13). Interestingly, frailty was not found to be associated with any other T cell subpopulation and thus, not with most memory T cell subpopulations. The results remained the same in a sensitivity analysis in which we restricted monocytes populations to be HLADR^+^. This showed that the associations found were not due to possible contamination of monocyte populations with NK cells and neutrophils (Table S5 and S6).

**Fig. 4:**
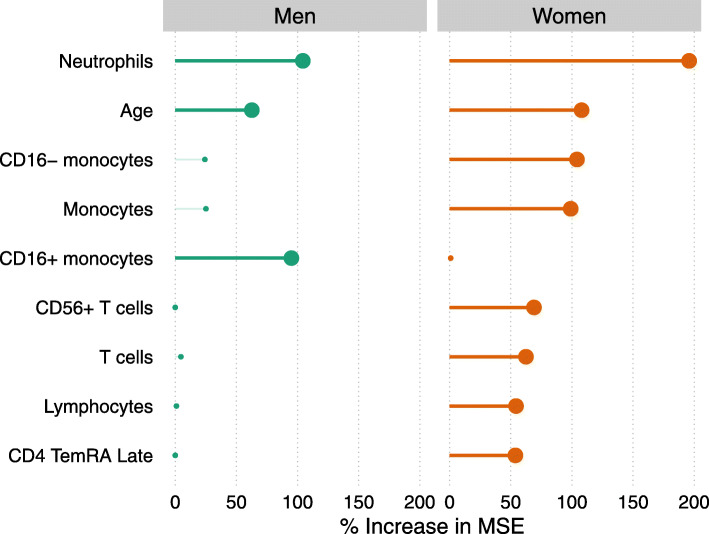
Variable importance plot showing the leukocyte subpopulations and other variables that are most helpful in predicting frailty in men (n=140) and women (n=137). Participants with missing frailty index score data (n=12) were excluded from analysis. The bold lines and dots represent the most important variables that show an increase in mean squared error (MSE) of >50%. See text for the meaning of variable importance

The prediction analysis, using the random forest algorithm, turned out to have low predictive power for frailty in men (5.5% PEV, i.e. proportion of explained variance). Therefore, it did not allow to confidently identify additional relations other than the ones found in the association study. The cell population highest in ranking of variable importance were neutrophils, closely followed by CD16^+^ monocytes (Fig. 4). In women however, the predictive accuracy was higher (23.1% PEV), implying that more of the relationships between cell populations and frailty could be confidently concluded to be ‘important’. Highest in ranking of variable importance in women were neutrophils followed by CD16- monocytes, total monocytes, CD56^+^ T cells, total T cells, lymphocytes and CD4 TemRA cells, thereby largely confirming the findings of the association study. In addition to these, relationships of frailty with total T cells and lymphocyte numbers were identified as being of similar importance as CD56^+^ T cells and CD4^+^ TemRA cells. Age also ranked high in both men and women. Noteworthy is that CMV serostatus was not important in the prediction models, with an increase in MSE of less than 7% in both sexes (data not shown).

**Fig. 5:**
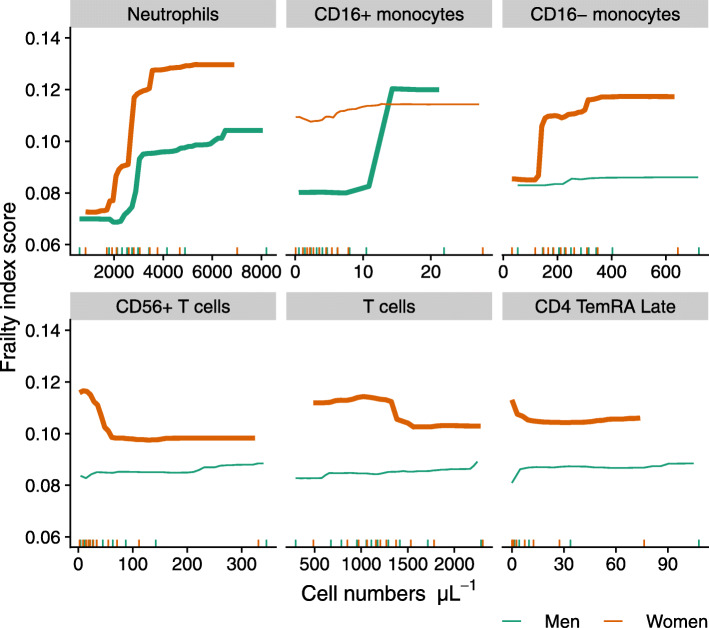
Partial dependence plots illustrating the role of the most important leukocyte phenotypes in predicting frailty in a random forest predictor for men (n=140) and women (n=137). Participants with missing frailty index score data (n=12) were excluded from analysis. The short vertical segments on the horizontal axis represent the deciles of the cell numbers in the data. Range of the figures is restricted to the part containing most of the data

An impression of the role of the more important variables in predicting frailty, both with regard to the strength and the direction of the effect, is provided by the partial dependence plots shown in Fig. 5 and Figure S4. These show the marginal ‘effect’ of a cell type on frailty, meaning the ‘effect’ of a cell type on frailty when the other variables in the random forest predictor are held constant at average values. The clearer and stronger relationships with frailty were seen in numbers of several myeloid cell populations (Fig. 5). The frailty index was higher with increasing neutrophil numbers in both men and women which was in accordance with the results of the association study. Numbers of classical monocytes (Fig. 5) and total monocytes (Figure S4) increased with frailty but only in women. Numbers of non-classical monocytes increased with frailty in men. However, this increase was only seen in the few participants with the highest (top 10%) CD16^+^ monocyte numbers (see Fig. 5 and its legend for explanation). In frailer women, a decrease was seen in numbers of CD56^+^ T cells, CD4^+^ TemRA cells, total T cells, and lymphocytes, although the marginal ‘effect’ was small. An explanation why the total numbers of T cells and lymphocytes in women were found to be of relevance in the random forest algorithm but not in the association study, may be found in interactions with other variables, which can be missed in an association study. To illustrate this, we investigated the joint relationship of total numbers of T cells and CD56^+^ T cells with frailty, which showed that the frailty score tended to be lowest when both total T cell numbers and CD56^+^ T cell numbers are high (Figure S5).

### Associations between frailty and proportions of leukocyte subpopulations

In addition, we investigated whether frailty was associated with relative values (percentages) of immune cell subpopulations within the major parent populations (that is, within T cells, B cells, monocytes, and NK cells). In this analysis, no associations with frailty were detected (Tables S5 and S6).

## Discussion

This study shows that frailty in a 60-87-year-old population was associated with higher absolute numbers of neutrophils in men and women, and also with several sex-specific changes in the immune cellular profile. To the best of our knowledge, this is the first study in which frailty was separately investigated in men and women for its relations with a wide variety of immune cell subpopulations. Frailer women demonstrated higher numbers of total- and CD16^-^ monocytes, lower numbers of CD56^+^ T cells, and lower numbers of late differentiated CD4^+^ TemRA T cells. These findings, obtained from association studies, were confirmed in a random forest prediction analysis. In men, the predictive value of the random forest analysis was too low to warrant substantial additional conclusions on top of the ones from the association study. The highest ranking subpopulation based on variable importance were neutrophils, in line with the association study. Based on variable importance, also CD16^+^ monocytes may play a role in prediction of frailty, but since this subpopulation was not found in the association study and the predictive accuracy was low, the relationship between CD16^+^ monocytes and frailty in men is unclear. In women, however, the predictive value of the random forest analysis was higher than in men and additional potentially relevant relationships were found with, respectively, total numbers of T cells and lymphocytes.

The associations found between higher neutrophil numbers and frailty are in line with previous reports [3, 5, 40]. Among the entire immune cellular profile we investigated, neutrophil numbers were most strongly associated with frailty and the association was found in both men and women. In a previous study it was shown that elevated neutrophil levels in humans are related to negative health outcomes [41], which corresponds to our results, since frail people are more prone to adverse outcomes like early mortality. The association of total monocyte numbers with frailty in women was also in line with other studies [3, 40]. We have now shown that the relationship of higher monocyte numbers with frailty in women was due to higher numbers of CD16^-^ classical monocytes. The classical CD16^-^ monocytes, having clear phagocytic capacity, further differentiate into macrophages [42], and are therefore involved in the defense against pathogens and in inflammation. Classical monocytes have a short lifespan of approximately one day in the circulation [43]. Thus, an increase in this population is likely due to an increased production by myeloid progenitor cells rather than due to reduced clearance.

Noteworthy is that the clearest associations with frailty found in our study were with myeloid cells. In a murine study, it was argued that both higher myeloid cell abundancies and lower vaccine responses in older mice could be the result of intrinsic differences in hematopoietic stem cells with age [44]. Other studies suggested that the myeloid bias of hematopoietic stem cell differentiation seen in old age is due to a chronic low-grade inflammation [45]. This stronger tendency of hematopoietic stem cells to differentiate in the myeloid direction in an environment of low-grade inflammation is possibly driven by plasma cells in the bone marrow that can produce pro-inflammatory cytokines which also influence myelopoiesis [46]. Such a chronic low-grade inflammation is often seen in frail people [47], with a role for the IL-6 pathway, since consistent associations have been reported between frailty and higher CRP levels [47]. Parallel to the more pronounced myeloid skewing with frailty in women, the association of chronic low-grade inflammation with frailty is also more pronounced in women than in men [3]. However, many other factors may impact cellular numbers in peripheral blood, like variations in extravascular homing, or differential apoptosis [48], which could explain the great variability between participants seen in our study population.

The present study also shows that several immune cell subpopulations differ in abundancy between the sexes, with higher numbers of most T cell subpopulations, especially CD4 T helper cells, and higher numbers of B cells, in women. These findings are in line with previous studies [25]. We also found sex-specific associations between frailty and lower numbers of several CD4 T cell subpopulations in women but not men, which has not been reported earlier. These results, together with the stronger associations between frailty and myeloid cell numbers in women, could suggest that the skewing of hematopoietic stem cells with frailty towards production of myeloid cells is more pronounced in women than in men. Immunological homeostasis in women may involve a different balance between CD4 and CD8 T cells than in men, with estrogen levels known to be involved in lymphocyte development and in particular CD4 T cell proliferation [49, 50]. In addition, immune function differs between the sexes, with women showing stronger responses to antigens and a stronger tendency to develop autoimmune diseases [49, 51]. Testosterone levels are also thought to impact this process, with testosterone possibly having an anti-inflammatory role [52]. The sex-specific findings in our study could also be of interest in relation to the so-called sex-frailty paradox, namely that women generally tend to be frailer, yet live longer than men [51]. In our study, frailer women had lower numbers of CD56^+^ T cells, which are known for their cytotoxic capacity [53]. While the relationship of frailty with CD56^+^ T cells has not been described previously, in one study NK cell markers (CD16 and CD56) were shown to be more highly expressed by T cells from people with better cognitive and physical functioning [54], which is in line with our findings. CD4^+^ TemRA T cell numbers were also found to be lower in frailer women. Since this was the weakest association in women, it would require confirmation in future studies. In fact, we expected to observe a negative correlation, i.e. meaning an increase of T cell memory numbers with frailty, instead of a lower number. It is known that the balance between naïve and memory T cells in peripheral blood changes with advancing age, mainly due to a decline in numbers of naïve (CD8^+^) T cells [11, 55, 56], which was also observed in our study (data not shown). Only few studies reported relationships between T cell populations and frailty and, to our knowledge, no associations were reported with late differentiated CD4^+^ TemRA T cells. In one study, frailty was related to lower naïve CD4^+^ T cells [8], in another one with lower CD4^+^ central memory (CD27^+^CD45RA^-^) T cells [6], while in a third study no association was found between T cell subpopulations and frailty [5]. Reasons for these heterogeneous results could arise from differences in gating strategies, not separating results for men and women, or not adjusting for CMV serostatus. Moreover, all previous studies involving memory T cell subpopulations were performed with relative values (percentages) rather than absolute cell numbers. Nevertheless, we found a relationship between frailty and lower numbers of total T cells in women. This relationship showed possible interactions with age and with numbers of CD56^+^ T cells, which could explain why it was detected in the random forest analysis but not in the association study.

In our study we confirmed previously reported associations of CMV seropositivity with the late-stage memory T cell population [11, 25]. We also observed that CMV seropositive men had lower classical monocyte numbers. In addition, their classical monocytes seemed to have higher CD38 expression. It is thought that CD38 can be upregulated in monocytes and macrophages in inflammatory conditions [21]. Further research is needed to confirm this association and to answer the question why it was only found in men. Furthermore, we observed that CMV serostatus was not important in our frailty prediction model. Conflicting evidence has been reported on whether there is a positive relationship between CMV serostatus and frailty, with some studies showing an association [57, 58] while others do not [5, 59]. These conflicting results might be explained by differences instruments used to evaluate frailty (frailty phenotype or frailty index), or differences in study population, with some studies being restricted to only one of the sexes [57] or to extreme elderly [5, 59].

A major strength of this study is the use of fresh whole blood samples to measure an extensive set of immune cell subpopulations. This made it possible to enumerate cell populations like neutrophils that cannot be quantified when using cryopreserved PBMCs. In addition, we were able to relate absolute numbers of 37 immune cell subpopulations to frailty. This is important as these are needed to correctly interpret relative values (e.g. percentages). For example, a change in relative values can be the result of a change in either the numerator or the denominator. This issue can be solved only with information on absolute numbers. Our study showed that, when we performed an analysis with subpopulations of B cells, T cells, NK cells, and monocytes expressed as percentages, this analysis did not give additional insight in how frailty relates to the immune cellular profile. Another strength of our study is the use of a robust statistical analysis framework with two different analytical approaches that complement each other to find associations with frailty while correcting for multiple testing. The analysis framework that we used performs well when there is a high number of explanatory variables and strong multicollinearity, as was the case in our study. In particular, the random forest algorithm is known for its accuracy in complex analyses when many explanatory variables are involved [60–62]. This algorithm made it possible to investigate how frailty is related to all cellular subpopulations together, i.e. the ‘cellular immune profile’. Of note is that we also used a quantitative measure of frailty that was defined in a consistent manner and implemented in a richly documented population, allowing the inclusion of 36 health characteristics. This made it possible to study the relation between frailty and immune cell subpopulations in a quantified manner. Lastly, our cohort consisted of a sample from the general community-dwelling population, thus allowing a window on aging of the immune system in a non-clinical setting. On the other hand, due to the nature of using fresh whole blood samples and our aim to quantify a large number of different immune cell subpopulations, we were restricted in the coverage of our antibody panel and therefore not all the subpopulations could be further classified with additional phenotypic markers. For example, to define regulatory T cells we used CD45RA and CD25 as was done previously [23–25], but not FoxP3 or CD127. In addition, it appeared, as already expected, that the variability in numbers of immune cell subpopulations is very large in older adults. This may have contributed to the low predictive accuracy observed in the prediction analysis. Furthermore, there is no consensus yet on which instrument is the best to investigate frailty, and different instruments might yield different results. Since we were interested in a measure that approximates general health, we used a Rockwood frailty index [16] based on 36 deficits rather than a frailty instrument with a less broad definition like the Fried frailty phenotype [63].

## Conclusions

The in-depth immune cellular profiling that we performed in this study showed that, among all investigated subpopulations, neutrophils were most strongly associated with frailty in men and women. In addition, sex-specific associations with frailty were revealed, with frailer women but not men showing higher numbers of classical (CD16^-^) monocytes and lower numbers of CD56^+^ T cells and late differentiated CD4^+^memory T cells than their healthier peers. An expected positive association between frailty and memory T cells was not observed. These results add to the evidence that frailty manifests differently in men and women. We hope that this will prompt further investigation into the different immune mechanisms associated with the development of frailty.

## Supplementary information

**Additional file 1** upplementary Figures S1-S5 and Tables S1-S8.

## Data Availability

The datasets used are available from the corresponding author upon reasonable request and with permission of the scientific committee of the Doetinchem Cohort Study.

## Abbreviations

CMV: Cytomegalovirus
DCS: Doetinchem cohort study
FDR: False discovery rate
Tem: Effector memory T cells
TemRA: Effector memory T cells re-expressing CD45RA
