# Supplementary material for: In-depth immune cellular profiling reveals sex-specific associations with frailty
Source: Immun Ageing. 2020 Jun 23;17:20. doi: 10.1186/s12979-020-00191-z (PMC7310472; doi:10.1186/s12979-020-00191-z)
Supplement: Supplementary file 1 — Additional file 1 upplementary Figures S1-S5 and Tables S1-S8. [file 12979_2020_191_MOESM1_ESM.pdf]

In-depth immune cellular profiling reveals sex-specific associations with frailty

Supplementary figures and tables

Leonard Daniël Samson, A. Mieke H. Boots, José A. Ferreira, H. Susan J. Picavet, Lia de Rond, Mary-lène de Zeeuw, W. M. Monique Verschuren, Anne-Marie Buisman, Peter Engelfriet

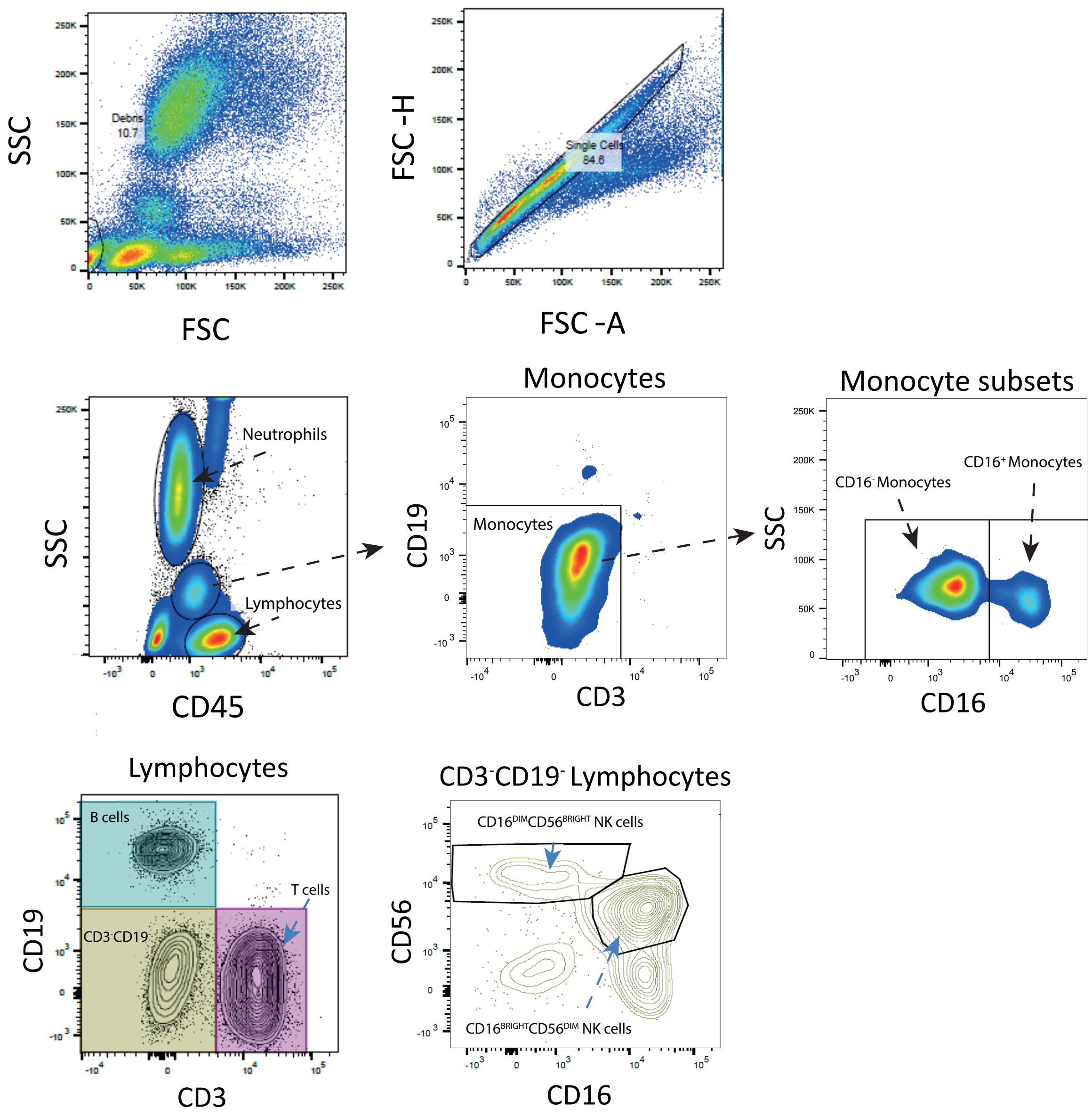

Figure S1: General gating strategy showing gating of single cells by FSC-A versus FSC-H, leukocyte subsets by SSC and CD45, monocyte subpopulations by CD16, T and B-cells by CD3 and CD19, and NK cells by CD16 and CD56.

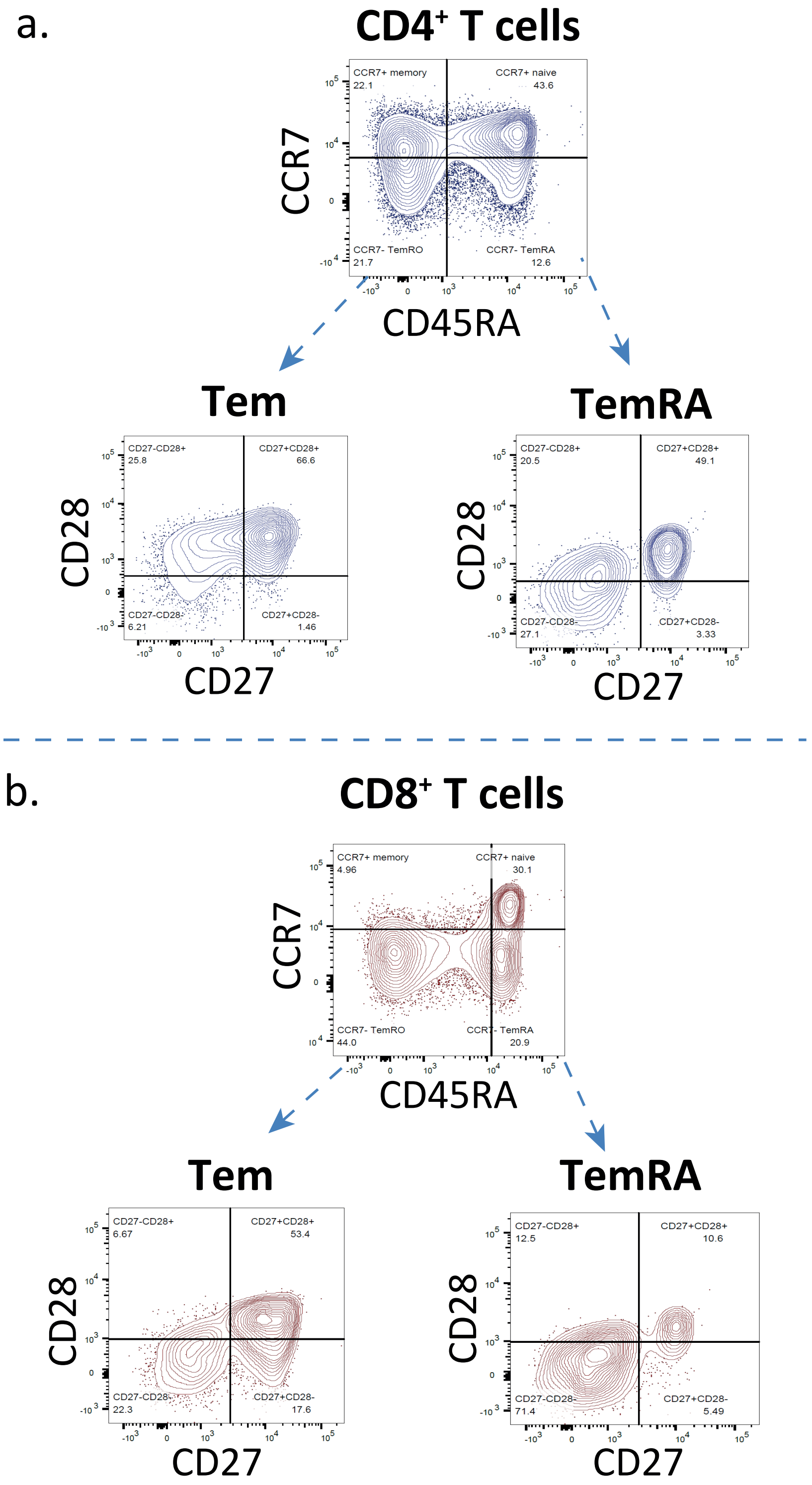

Figure S2: Gating strategy for (a) memory CD4 T cells and (b) memory CD8 T cells. Tem: effector memory (CD4 or CD8 positive) T cells. TemRA: Terminally differentiated (CD4 or CD8 positive) T cells re-expressing CD45RA.

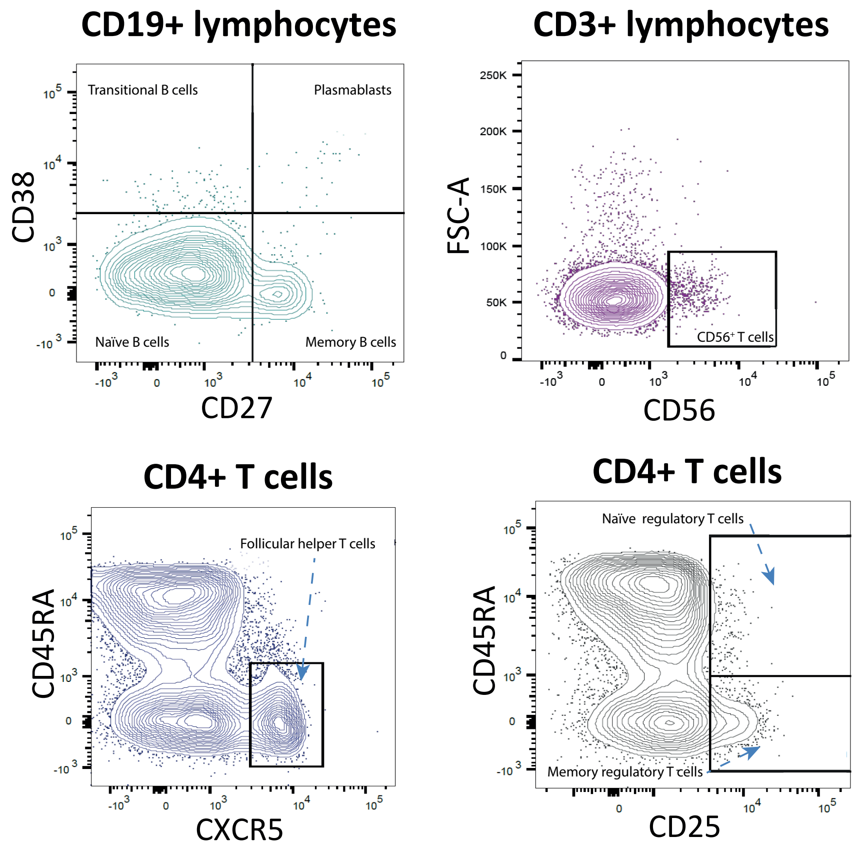

Figure S3: Gating strategy for (a) B cell subsets and (b) follicular helper T cells and regulatory T cells.

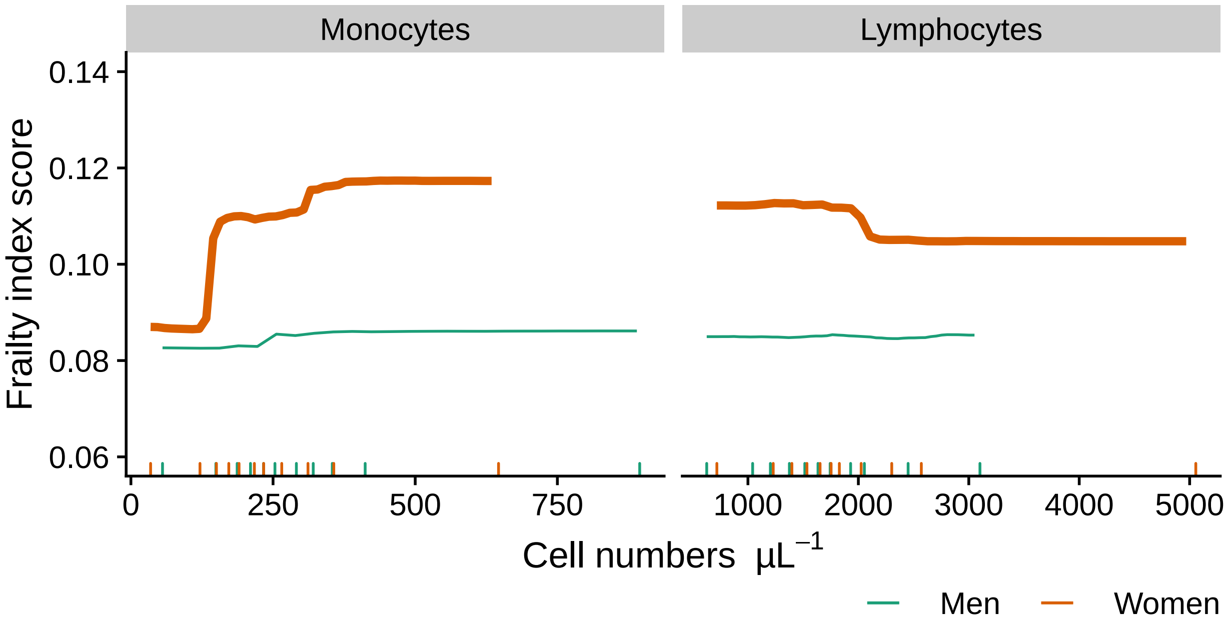

Figure S4: Partial dependence plots showing how frailty 'depends' on total monocyte numbers and total lymphocyte numbers in men (n=140) and women (n=137). Participants with missing frailty index score data (n=12) were excluded from analysis. The short vertical segments on the horizontal axis represent the deciles of the cell numbers in the data. Range of the figures is restricted to the part containing most of the data.

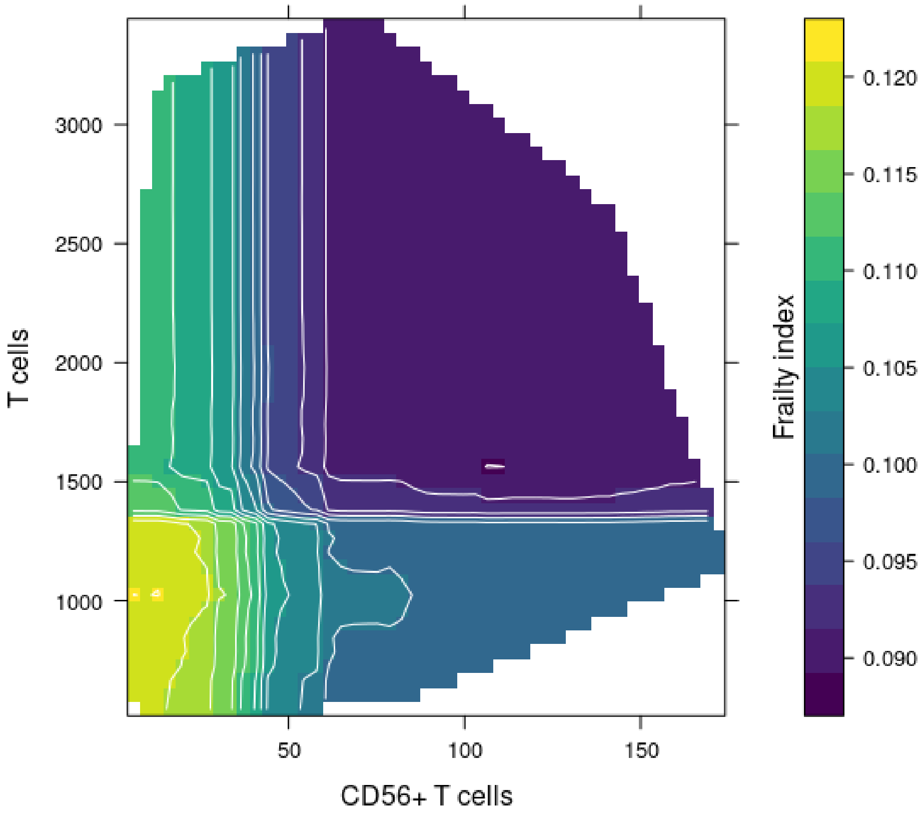

Figure S5: Partial dependence plot with the possible joint relationship of total T cell numbers and CD56$^+$ T cell numbers on frailty. To avoid extrapolation, the plot is restricted to the area of which data is available.

Table S1: Definition of cell phenotypes by expression of cell surface markers

Cell phenotype	Definition
**T cells**	
T cells	$CD45^{+}CD3^{+}$
T Follicular helper	$CD3^{+}CD4^{+}CD45RA^{-}CXCR5^{+}$
$CD56^{+}$ T cells	$CD45^{+}CD3^{+}CD56^{+}$
CD4/CD8 ratio	CD4 T cells/CD8 T cells
CD4 T cells	$CD45^{+}CD3^{+}CD4^{+}$
CD4 Naive	$CD3^{+}CD4^{+}CD45RA^{+}CCR7^{+}$
CD4 CM	$CD3^{+}CD4^{+}CD45RA^{-}CCR7^{+}$
CD4 TemRA	$CD3^{+}CD4^{+}CD45RA^{+}CCR7^{-}$
CD4 TemRA Early	$CD3^{+}CD4^{+}CD45RA^{+}CCR7^{-}CD27^{+}CD28^{+}$
CD4 TemRA Late	$CD3^{+}CD4^{+}CD45RA^{+}CCR7^{-}CD27^{-}CD28^{-}$
CD4 Tem	$CD3^{+}CD4^{+}CD45RA^{-}CCR7^{-}$
CD4 Tem Early	$CD3^{+}CD4^{+}CD45RA^{-}CCR7^{-}CD27^{+}CD28^{+}$
CD4 Tem Late	$CD3^{+}CD4^{+}CD45RA^{-}CCR7^{-}CD27^{-}CD28^{-}$
Regulatory T cells	$CD3^{+}CD4^{+}CD25^{BRIGHT}$
Treg naive	$CD3^{+}CD4^{+}CD25^{BRIGHT}CD45RA^{+}$
Treg memory	$CD3^{+}CD4^{+}CD25^{BRIGHT}CD45RA^{-}$
CD8 T cells	$CD45^{+}CD3^{+}CD8^{+}$
CD8 Naive	$CD3^{+}CD8^{+}CD45RA^{+}CCR7^{+}$
CD8 CM	$CD3^{+}CD8^{+}CD45RA^{-}CCR7^{+}$
CD8 TemRA	$CD3^{+}CD8^{+}CD45RA^{+}CCR7^{-}$
CD8 TemRA Early	$CD3^{+}CD8^{+}CD45RA^{+}CCR7^{-}CD27^{+}CD28^{+}$
CD8 TemRA Late	$CD3^{+}CD8^{+}CD45RA^{+}CCR7^{-}CD27^{-}CD28^{-}$
CD8 Tem	$CD3^{+}CD8^{+}CD45RA^{-}CCR7^{-}$
CD8 Tem Early	$CD3^{+}CD8^{+}CD45RA^{-}CCR7^{-}CD27^{+}CD28^{+}$
CD8 Tem Late	$CD3^{+}CD8^{+}CD45RA^{-}CCR7^{-}CD27^{-}CD28^{-}$
**Neutrophils**	
Neutrophils	$SSC^{BRIGHT}CD45^{DIM}$
Neutrophils, CD16 expr.	$SSC^{BRIGHT}CD45^{DIM}$;$CD16^{expression}$
**Monocytes**	
Monocytes	$SSC^{DIM}CD45^{+}CD3^{-}CD19^{-}$
$CD16^{-}$ monocytes	$SSC^{DIM}CD45^{+}CD16^{-}$
$CD16^{-}$ mon. CD38 expr.	$SSC^{DIM}CD45^{+}CD16^{-}$;$CD38^{expression}$
$CD16^{-}$ mon. HLADR expr.	$SSC^{DIM}CD45^{+}CD16^{-}$;$HLADR^{expression}$
$CD16^{+}$ monocytes	$SSC^{DIM}CD45^{+}CD16^{+}$
$CD16^{+}$ mon. CD38 expr.	$SSC^{DIM}CD45^{+}CD16^{+}$;$CD38^{expression}$
$CD16^{+}$ mon. HLADR expr.	$SSC^{DIM}CD45^{+}CD16^{+}$;$HLADR^{expression}$
**B cells**	
B cells	$CD45^{+}CD19^{+}$
Plasmablasts	$CD45^{+}CD19^{+}CD38^{BRIGHT}CD27^{BRIGHT}$
Transitional B cells	$CD45^{+}CD19^{+}CD38^{BRIGHT}CD27^{-}$
Naive B cells	$CD45^{+}CD19^{+}CD38^{DIM}CD27^{-}$
Memory B cells	$CD45^{+}CD19^{+}CD38^{DIM}CD27^{+}$
**NK cells**	
NK cells	Either NK $CD56^{BRIGHT}$ or NK $CD56^{DIM}$
NK $CD56^{BRIGHT}$	$CD45^{+}CD3^{-}CD19^{-}CD16^{DIM}CD56^{BRIGHT}$
NK $CD56^{DIM}$	$CD45^{+}CD3^{-}CD19^{-}CD16^{BRIGHT}CD56^{DIM}$

Table S2: Leukocyte numbers per sex and CMV serostatus

	Men			Women		
	CMV- (n=69)	CMV+ (n=76)	P value[1]	CMV- (n=53)	CMV+ (n=91)	P value[1]
**T cells**						
T cells	1002.6 (414.8)	1203.5 (481.2)	<0.001*	1124.3 (427.2)	1270.8 (433.6)	0.026*
T Follicular helper	49.5 (28.3)	62 (32.9)	0.04*	65.9 (35.2)	67.4 (40.3)	0.374
$CD56^+$ T cells	21.8 (30.7)	63.3 (111.5)	<0.001*	16.2 (16.6)	47.4 (66.6)	<0.001*
CD4/CD8 ratio	3.9 (2.7)	2.5 (2)	0.002*	4.2 (3.2)	2.8 (1.6)	<0.001*
CD4 T cells	683.1 (369.1)	769.1 (424.1)	0.041*	896 (367.6)	890 (337.4)	0.847
CD4 Naive	321.9 (267.5)	255.6 (216)	0.292	343.7 (262.5)	372.5 (304.6)	0.924
CD4 CM	175.6 (91)	217.5 (95.8)	0.01*	234.1 (130.2)	231.3 (146.9)	0.179
CD4 TemRA	27.9 (29.5)	48.6 (44.5)	<0.001*	33.9 (51.5)	53 (57.1)	0.003*
CD4 TemRA Early	22.6 (27.4)	20.2 (21)	0.666	29.7 (43)	27.9 (31.9)	0.839
CD4 TemRA Late	0.1 (0.3)	5.8 (26.6)	<0.001*	0.1 (0.2)	5.9 (14.2)	<0.001*
CD4 Tem	110.6 (70.1)	159.1 (116.2)	<0.001*	151.3 (86.2)	176.7 (113.2)	0.177
CD4 Tem Early	77.7 (51.4)	89.2 (55.8)	0.166	114.1 (60.4)	104 (74.8)	0.362
CD4 Tem Late	1.1 (1.5)	15.3 (28)	<0.001*	1.1 (1.6)	9 (14.3)	<0.001*
Regulatory T cells	34.7 (18.8)	34.8 (23.6)	0.556	42.6 (22.4)	42.4 (24.2)	0.377
Treg naive	6.8 (4)	7.2 (3.2)	0.607	8.3 (5.9)	10.1 (5.7)	0.479
Treg memory	9.3 (5.9)	10.2 (5.2)	0.343	10.5 (5.5)	9.1 (6.4)	0.049
CD8 T cells	188.9 (199.8)	337.3 (247.2)	<0.001*	199.6 (129.5)	330.7 (162.4)	<0.001*
CD8 Naive	20.9 (31.9)	18 (28.4)	0.532	36.7 (45.4)	36 (43.4)	0.948
CD8 CM	13.7 (11.3)	17.6 (15.7)	0.01*	17.3 (13.3)	16.9 (15.7)	0.76
CD8 TemRA	32.5 (42.2)	84.6 (108.9)	<0.001*	27.8 (26.6)	74.2 (72)	<0.001*
CD8 TemRA Early	6.8 (7.7)	7.4 (8.4)	0.73	6.2 (7.4)	9.2 (10.3)	0.129
CD8 TemRA Late	10.5 (15.5)	54.8 (65.1)	<0.001*	8.6 (12.6)	39.9 (49.2)	<0.001*
CD8 Tem	120.1 (121.6)	229.4 (140)	<0.001*	115.8 (62.6)	210 (109.4)	<0.001*
CD8 Tem Early	64.3 (64.1)	73.3 (71)	0.409	74.4 (52.2)	89.5 (65.3)	0.538
CD8 Tem Late	11.4 (14.7)	58.9 (71)	<0.001*	6.6 (7.5)	47.5 (52.7)	<0.001*
**Neutrophils**						
Neutrophils	2811.5 (2202.7)	2646 (1236.8)	0.197	2681 (1451.6)	2819.5 (1559.9)	0.733
Neutrophils, CD16 expr.[2]	3671 (5693)	4032 (5797)	0.393	4509 (5447)	4389 (5471)	0.86
**Monocytes**						
Monocytes	276.8 (133)	247.4 (122.4)	0.057*	233.4 (144)	208.6 (120.7)	0.365
$CD16^-$ monocytes	273.6 (137.1)	246.1 (118.1)	0.064*	229.3 (144.7)	206.1 (120.2)	0.351
$CD16^-$ mon. CD38 expr.[2]	1190 (336)	1266.5 (339.5)	0.039*	1168 (190.5)	1225 (291.2)	0.401
$CD16^-$ mon. HLADR expr.[2]	8953 (6199)	8716.5 (4564.2)	0.78	8834 (3573.5)	9085 (6528)	0.907
$CD16^+$ monocytes	4 (4.7)	4 (4.1)	0.397	4 (3.8)	3.4 (3.9)	0.628
$CD16^+$ mon. CD38 expr.[2]	447 (199)	483 (197)	0.157	407 (143.5)	449 (219)	0.138
$CD16^+$ mon. HLADR expr.[2]	57197 (28277)	52852 (41112.2)	0.587	42036 (38598.5)	40301 (36045.5)	0.935
**B cells**						
B cells	134.1 (87.2)	151.6 (103.7)	0.427	167.7 (114)	171.3 (118.2)	0.904
Plasmablasts	1.4 (1.3)	1.4 (1.2)	0.677	1.5 (1.5)	1.4 (1.2)	0.644
Transitional B cells	3.5 (3.8)	4.1 (5.5)	0.643	4.5 (5.2)	4.2 (4.4)	0.806
Naive B cells	93.6 (71.4)	112.5 (74.9)	0.487	124.8 (89.6)	120.1 (79.3)	0.826
Memory B cells	25.1 (24.6)	27.7 (29.9)	0.73	31.1 (31.4)	36.5 (33.6)	0.667
**NK cells**						
NK cells	276.2 (216.4)	217.3 (194.2)	0.109	227.5 (115.8)	206.5 (140.6)	0.39
NK $CD56^{BRIGHT}$	11.2 (7.3)	10.3 (7.8)	0.872	11.6 (8.7)	9.7 (6.9)	0.199
NK $CD56^{DIM}$	256.5 (216.5)	199 (181.7)	0.116	219.7 (106.4)	196.3 (141.4)	0.46

Note:
All values are median (interquartile range) cell numbers per µL, unless otherwise stated
[1] P values of Kruskal-Wallis rank sum test between CMV serostatus per sex, adjusted for age
[2] Values in Median fluoresence intensity
* Selected outcomes when the False Discovery Rate is set to a maximum of 15%

Table S3: Spearman associations between immune cell subpopulations and frailty in men (n=140)

Immune cell subset	Spearman's rho	P value	FDR*
**Neutrophils**	**0.25**	**0.002**	**0.077**
Transitional B cells	-0.19	0.015	0.324
Monocytes	0.17	0.027	0.389
$CD16^-$ monocytes	0.16	0.036	0.383
CD4 Naive	0.17	0.040	0.341
CD4 TemRA Late	0.13	0.078	0.562
CD4 TemRA	0.17	0.090	0.553
Regulatory T cells	0.12	0.104	0.561
$CD16^+$ monocytes	0.16	0.133	0.634
$CD16^-$ mon. HLADR expr.	-0.15	0.133	0.571
NK $CD56^{DIM}$	-0.08	0.172	0.672
CD4 TemRA Early	0.14	0.191	0.686
CD4 T cells	0.09	0.207	0.683
NK cells	-0.06	0.208	0.638
CD4/CD8 ratio	0.10	0.219	0.628
Memory B cells	-0.07	0.319	0.857
T cells	0.09	0.321	0.812
CD8 Tem Early	-0.07	0.354	0.844
Treg naive	0.08	0.387	0.877
Neutrophils, CD16 expr.	-0.10	0.399	0.858
T Follicular helper	0.07	0.439	0.898
$CD16^+$ mon. CD38 expr.	-0.05	0.440	0.860
Lymphocytes	0.04	0.574	1.073
NK $CD56^{BRIGHT}$	-0.03	0.582	1.042
CD8 Naive	0.05	0.590	1.014
CD8 CM	-0.05	0.650	1.074
CD8 T cells	0.03	0.658	1.048
B cells	-0.06	0.667	1.025
CD8 Tem Late	0.09	0.689	1.022
$CD56^+$ T cells	0.03	0.694	0.995
CD8 Tem	0.02	0.697	0.967
$CD16^-$ mon. CD38 expr.	-0.01	0.705	0.948
$CD16^+$ mon. HLADR expr.	0.07	0.728	0.949
CD4 Tem Late	0.03	0.735	0.930
CD4 CM	-0.05	0.757	0.931
CD4 Tem Early	-0.03	0.789	0.943
CD8 TemRA Late	0.02	0.796	0.925
Plasmablasts	0.04	0.810	0.916
Naive B cells	-0.05	0.839	0.925
CD8 TemRA	0.02	0.851	0.915
CD8 TemRA Early	-0.01	0.931	0.976
Treg memory	0.03	0.967	0.990
CD4 Tem	0.00	0.994	0.994

Note:
The (Spearman) associations are ordered by p value, with the lowest p values shown at the top. Associations that were selected with a FDR lower than 15% are shown in bold.

* False Discovery Rate (estimated)

Table S4: Spearman associations between immune cell subpopulations and frailty in women (n=137)

Immune cell subset	Spearman's rho	P value	FDR*
**Neutrophils**	**0.40**	**<0.001**	**0.000**
**CD16⁻ monocytes**	**0.24**	**0.003**	**0.066**
**Monocytes**	**0.23**	**0.004**	**0.054**
**CD56+ T cells**	**-0.20**	**0.01**	**0.109**
**CD4 TemRA Late**	**-0.13**	**0.015**	**0.128**
CD16- mon. CD38 expr.	-0.20	0.022	0.157
CD4 Tem Early	0.20	0.032	0.194
CD16+ mon. CD38 expr.	-0.18	0.037	0.200
CD16- mon. HLADR expr.	-0.12	0.063	0.302
CD4 Tem	0.15	0.073	0.313
NK CD56^{BRIGHT}	-0.15	0.08	0.311
CD8 TemRA	-0.09	0.091	0.325
Transitional B cells	-0.16	0.096	0.318
CD8 TemRA Late	-0.08	0.099	0.303
NK CD56^{DIM}	-0.15	0.133	0.382
NK cells	-0.13	0.149	0.401
CD8 CM	0.14	0.165	0.418
CD4 Tem Late	-0.04	0.235	0.562
CD4/CD8 ratio	0.11	0.25	0.567
CD4 TemRA Early	0.09	0.274	0.589
CD8 T cells	-0.10	0.322	0.658
CD4 CM	0.07	0.34	0.665
CD4 Naive	-0.11	0.344	0.643
T Follicular helper	0.09	0.347	0.621
CD8 Naive	-0.13	0.359	0.618
Treg memory	0.07	0.374	0.618
CD8 TemRA Early	-0.02	0.422	0.672
Memory B cells	-0.09	0.428	0.658
Plasmablasts	-0.07	0.461	0.683
T cells	-0.05	0.49	0.702
CD8 Tem Early	0.12	0.519	0.720
Lymphocytes	-0.02	0.526	0.707
CD16+ monocytes	0.07	0.551	0.718
B cells	-0.06	0.579	0.732
Neutrophils, CD16 expr.	-0.01	0.73	0.897
Treg naive	-0.04	0.779	0.930
CD8 Tem	0.02	0.814	0.946
CD4 TemRA	0.00	0.913	1.033
CD16+ mon. HLADR expr.	0.01	0.946	1.044
CD8 Tem Late	0.01	0.953	1.025
Regulatory T cells	0.01	0.966	1.013
CD4 T cells	0.01	0.967	0.990
Naive B cells	0.00	0.984	0.984

Note:
The (Spearman) associations are ordered by p value, with the lowest p values shown at the top. Associations that were selected with a FDR lower than 15% are shown in bold.

* False Discovery Rate (estimated)

Table S5: Repeated sensitivity analysis in men when monocytes were restricted to be HLADR+

Immune cell subset	Spearman's rho	P value	FDR*
**Neutrophils**	**0.25**	**0.002**	**0.061**
Transitional B cells	-0.19	0.014	0.276
Monocytes (HLADR^{+})	0.17	0.027	0.349
CD4 Naive	0.17	0.040	0.386
CD16^{-} monocytes (HLADR^{+})	0.14	0.048	0.378
CD4 TemRA Late	0.13	0.078	0.508
CD4 TemRA	0.17	0.091	0.508
Regulatory T cells	0.12	0.104	0.508
CD16^{+} monocytes (HLADR^{+})	0.17	0.108	0.470
NK CD56^{DIM}	-0.08	0.172	0.671
CD4 TemRA Early	0.14	0.191	0.677
CD4 T cells	0.09	0.205	0.666
NK cells	-0.06	0.209	0.626
CD4/CD8 ratio	0.10	0.222	0.617
T cells	0.09	0.319	0.828
Memory B cells	-0.07	0.320	0.780
CD8 Tem Early	-0.07	0.354	0.812
Treg naive	0.08	0.390	0.844
Neutrophils, CD16 expr.	-0.10	0.401	0.822
T Follicular helper	0.07	0.441	0.860
Lymphocytes	0.04	0.574	1.066
NK CD56^{BRIGHT}	-0.03	0.580	1.029
CD8 Naive	0.05	0.589	0.998
CD8 CM	-0.05	0.651	1.058
CD8 T cells	0.03	0.657	1.026
B cells	-0.06	0.669	1.003
CD8 Tem Late	0.09	0.688	0.994
CD56^{+} T cells	0.03	0.695	0.968
CD8 Tem	0.02	0.697	0.938
CD4 Tem Late	0.03	0.734	0.954
CD4 CM	-0.05	0.756	0.951
CD4 Tem Early	-0.03	0.789	0.962
CD8 TemRA Late	0.02	0.794	0.938
Plasmablasts	0.04	0.807	0.926
Naive B cells	-0.05	0.838	0.934
CD8 TemRA	0.02	0.853	0.924
CD8 TemRA Early	-0.01	0.929	0.980
Treg memory	0.03	0.966	0.992
CD4 Tem	0.00	0.994	0.994

Note:
The (Spearman) associations are ordered by p value, with the lowest p values shown at the top. Associations that were selected with a FDR lower than 15% are shown in bold

* False Discovery Rate (estimated)

Table S6: Repeated sensitivity analysis in women when monocytes were restricted to be HLADR+

Immune cell subset	Spearman's rho	P value	FDR*
**Neutrophils**	**0.40**	**$<$0.001**	**0.000**
**Monocytes (HLADR$^+$)**	**0.23**	**0.004**	**0.074**
**CD56$^+$ T cells**	**-0.20**	**0.01**	**0.124**
**CD16$^-$ monocytes (HLADR$^+$)**	**0.18**	**0.014**	**0.139**
**CD4 TemRA Late**	**-0.13**	**0.015**	**0.120**
CD4 Tem Early	0.20	0.032	0.208
CD4 Tem	0.15	0.071	0.395
NK CD56^{BRIGHT}	-0.15	0.078	0.380
CD8 TemRA	-0.09	0.09	0.390
Transitional B cells	-0.16	0.096	0.375
CD8 TemRA Late	-0.08	0.099	0.350
NK CD56^{DIM}	-0.15	0.133	0.433
NK cells	-0.13	0.149	0.446
CD8 CM	0.14	0.167	0.466
CD4 Tem Late	-0.04	0.235	0.610
CD4/CD8 ratio	0.11	0.251	0.612
CD4 TemRA Early	0.09	0.274	0.628
CD8 T cells	-0.10	0.324	0.701
CD4 CM	0.07	0.34	0.697
T Follicular helper	0.09	0.345	0.674
CD4 Naive	-0.11	0.346	0.642
CD8 Naive	-0.13	0.359	0.637
Treg memory	0.07	0.375	0.636
CD8 TemRA Early	-0.02	0.423	0.688
Memory B cells	-0.09	0.428	0.668
CD16$^+$ monocytes (HLADR$^+$)	0.09	0.441	0.661
Plasmablasts	-0.07	0.458	0.662
T cells	-0.05	0.49	0.683
CD8 Tem Early	0.12	0.521	0.701
Lymphocytes	-0.02	0.528	0.686
B cells	-0.06	0.578	0.727
Neutrophils, CD16 expr.	-0.01	0.729	0.888
Treg naive	-0.04	0.78	0.921
CD8 Tem	0.02	0.814	0.934
CD4 TemRA	0.00	0.914	1.018
CD8 Tem Late	0.01	0.953	1.032
Regulatory T cells	0.01	0.965	1.017
CD4 T cells	0.01	0.967	0.992
Naive B cells	0.00	0.985	0.985

Note:
The (Spearman) associations are ordered by p value, with the lowest p values shown at the top. Associations that were selected with a FDR lower than 15% are shown in bold

* False Discovery Rate (estimated)

Table S7: Additional analysis in men (n=140) to test associations with frailty when percentages of subpopulations are used instead of absolute numbers

Immune cell subset	Spearman's rho	P value	FDR*
Transitional B cells, (%)	-0.22	0.011	0.373
CD4 Naive, (%)	0.22	0.018	0.302
CD4 CM, (%)	-0.24	0.023	0.260
T cells, (%)	0.16	0.047	0.395
NK cells, (%)	-0.11	0.091	0.621
CD4 TemRA Late, (%)	0.11	0.169	0.958
B cells, (%)	-0.14	0.181	0.880
NK $CD56^{BRIGHT}$, (%)	0.10	0.185	0.787
CD4 Tem, (%)	-0.10	0.186	0.702
CD4 Tem Early, (%)	-0.11	0.204	0.695
CD8 T cells, (%)	-0.11	0.209	0.644
CD4 T cells, (%)	0.12	0.213	0.603
Treg memory, (%)	-0.09	0.215	0.562
NK $CD56^{DIM}$, (%)	-0.08	0.247	0.600
CD4 TemRA, (%)	0.10	0.260	0.589
CD8 Naive, (%)	0.08	0.342	0.727
Regulatory T cells, (%)	0.05	0.425	0.849
CD8 CM, (%)	-0.03	0.464	0.877
CD8 Tem Early, (%)	-0.07	0.469	0.840
CD8 Tem Late, (%)	0.11	0.474	0.806
CD4 TemRA Early, (%)	0.06	0.573	0.927
Plasmablasts, (%)	0.04	0.582	0.900
Memory B cells, (%)	-0.01	0.670	0.990
CD8 TemRA, (%)	0.03	0.676	0.958
Naive B cells, (%)	0.01	0.705	0.958
CD8 TemRA Late, (%)	0.00	0.719	0.940
CD8 TemRA Early, (%)	0.00	0.769	0.969
T Follicular helper, (%)	-0.03	0.793	0.963
$CD16^-$ monocytes, (%)	0.01	0.838	0.983
CD4 Tem Late, (%)	-0.05	0.848	0.961
CD8 Tem, (%)	0.08	0.865	0.948
$CD16^+$ monocytes, (%)	-0.01	0.890	0.945
Treg naive, (%)	-0.02	0.934	0.962
$CD56^+$ T cells, (%)	-0.03	0.992	0.992

Note:
The (Spearman) associations are ordered by p value, with the lowest p values shown at the top. Associations that were selected with a FDR lower than 15% are shown in bold

* False Discovery Rate (estimated)

Table S8: Additional analysis in women (n=137) to test associations with frailty when percentages of sub-populations are used instead of absolute numbers

Immune cell subset	Spearman's rho	P value	FDR*
$CD56^+$ T cells, (%)	-0.18	0.009	0.304
CD4 TemRA Late, (%)	-0.17	0.009	0.156
CD8 Tem, (%)	0.20	0.021	0.236
Transitional B cells, (%)	-0.21	0.022	0.188
CD4 Tem Early, (%)	0.17	0.029	0.195
CD8 Tem Early, (%)	0.18	0.056	0.319
CD8 CM, (%)	0.14	0.062	0.301
CD8 TemRA Late, (%)	-0.09	0.065	0.275
CD4 Tem, (%)	0.16	0.066	0.251
CD8 TemRA, (%)	-0.09	0.075	0.256
Naive B cells, (%)	0.13	0.086	0.264
NK cells, (%)	-0.17	0.093	0.264
CD4 Naive, (%)	-0.12	0.133	0.347
CD4 CM, (%)	0.14	0.138	0.336
CD4 T cells, (%)	0.10	0.205	0.465
Treg memory, (%)	0.07	0.229	0.486
CD4 Tem Late, (%)	-0.05	0.232	0.465
CD4 TemRA Early, (%)	0.10	0.258	0.487
T cells, (%)	0.16	0.293	0.524
CD8 T cells, (%)	-0.09	0.294	0.499
Memory B cells, (%)	-0.08	0.334	0.540
T Follicular helper, (%)	0.10	0.350	0.540
$CD16^+$ monocytes, (%)	-0.02	0.458	0.677
Treg naive, (%)	-0.05	0.460	0.651
CD8 Tem Late, (%)	0.09	0.480	0.653
$CD16^-$ monocytes, (%)	0.07	0.547	0.716
B cells, (%)	-0.06	0.572	0.720
CD8 Naive, (%)	-0.10	0.662	0.804
Plasmablasts, (%)	-0.04	0.694	0.814
Regulatory T cells, (%)	-0.04	0.726	0.823
CD8 TemRA Early, (%)	-0.04	0.728	0.798
NK $CD56^{DIM}$, (%)	0.02	0.825	0.877
NK $CD56^{BRIGHT}$, (%)	-0.02	0.825	0.850
CD4 TemRA, (%)	0.00	0.893	0.893

Note:
The (Spearman) associations are ordered by p value, with the lowest p values shown at the top. Associations that were selected with a FDR lower than 15% are shown in bold

* False Discovery Rate (estimated)
